# Time-dependent optimized coupled-cluster method with doubles and perturbative triples for first principles simulation of multielectron dynamics

**DOI:** 10.3389/fchem.2022.982120

**Published:** 2022-09-13

**Authors:** Himadri Pathak, Takeshi Sato, Kenichi L. Ishikawa

**Affiliations:** ^1^ Department of Nuclear Engineering and Management, School of Engineering, The University of Tokyo, Tokyo, Japan; ^2^ Photon Science Center, School of Engineering, The University of Tokyo, Tokyo, Japan; ^3^ Research Institute for Photon Science and Laser Technology, The University of Tokyo, Tokyo, Japan

**Keywords:** multielectron dynamics, time-dependent optimized coupled-cluster, high harmonic generation, strong laser field, strong field ionization

## Abstract

We report the formulation of a new, cost-effective approximation method in the time-dependent optimized coupled-cluster (TD-OCC) framework [T. Sato *et al.*, J. Chem. Phys. 148, 051101 (2018)] for first-principles simulations of multielectron dynamics in an intense laser field. The method, designated as TD-OCCD(T), is a time-dependent, orbital-optimized extension of the “gold-standard” CCSD(T) method in the ground-state electronic structure theory. The equations of motion for the orbital functions and the coupled-cluster amplitudes are derived based on the real-valued time-dependent variational principle using the fourth-order Lagrangian. The TD-OCCD(T) is size extensive and gauge invariant, and scales as *O*(*N* ^7^) with respect to the number of active orbitals *N*. The pilot application of the TD-OCCD(T) method to the strong-field ionization and high-order harmonic generation from a Kr atom is reported in comparison with the results of the previously developed methods, such as the time-dependent complete-active-space self-consistent field (TD-CASSCF), TD-OCC with double and triple excitations (TD-OCCDT), TD-OCC with double excitations (TD-OCCD), and the time-dependent Hartree-Fock (TDHF) methods.

## 1 Introduction

Recent years witnessed unprecedented progress in laser technologies, which made it possible to observe the motions of electrons at the attosecond time scale (Itatani et al. (2004); Corkum and Krausz (2007); Krausz and Ivanov (2009); Baker et al. (2006)). On the other hand, various theoretical and numerical methods have been developed for interpreting, understanding, and predicting the experiments.

The multi-configuration time-dependent Hartree-Fock (MCTDHF) method (Caillat et al. (2005); Kato and Kono (2004); Nest et al. (2005); Haxton et al. (2011); Hochstuhl and Bonitz (2011)), and the time-dependent complete-active-space self-consistent-field (TD-CASSCF) method (Sato and Ishikawa (2013); Sato et al. (2016); Sato et al. (2018a)) are the most rigorous approaches to solve time-dependent Schrödinger equation (TDSE) of many-electron systems, where the wavefunction is given by the full configuration interaction (FCI) expansion,

$$\Psi \left(t\right)=\underset{I}{\sum }C_{I}\left(t\right)\Phi_{I}\left(t\right),$$ (1)

with both CI coefficients {*C*_***I***_(*t*)} and orbital functions {*ψ*_*p*_(*t*)} constituting Slater determinants {Φ_***I***_(*t*)} are propagated in time according to the time-dependent variational principle (TDVP). The TD-CASSCF method broadens the applicability of the MCTDHF method by flexibly classifying the orbital subspace into frozen-core, dynamical-core, and active. Unfortunately, the factorial computational scaling impedes large-scale applications. There are reports of various affordable size-inextensive methods (Miyagi and Madsen (2013, 2014); Haxton and McCurdy (2015); Sato and Ishikawa (2015)) developed by limiting the CI expansion of the wavefunction. Alternatively, the size-extensive coupled-cluster method, which relies on an exponential wavefunction, is a superior choice to address these problems with a polynomial cost-scaling (Kümmel (2003); Shavitt and Bartlett (2009)). We have developed an explicitly time-dependent coupled-cluster method considering optimized orthonormal orbitals within the flexibly chosen active space, called the time-dependent optimized coupled-cluster (TD-OCC) method, (Sato et al. (2018b)) including double (TD-OCCD) and double and triple excitation amplitudes (TD-OCCDT). Our method is a time-dependent formulation of the stationary optimized coupled-cluster method (Scuseria and Schaefer (1987); Sherrill et al. (1998); Krylov et al. (1998)). Kvaal (Kvaal (2012)) also developed an orbital adaptive time-dependent coupled-cluster (OATDCC) method using biorthogonal orbitals. We take note of a few reports on the time-dependent coupled-cluster methods (Huber and Klamroth (2011); Pigg et al. (2012); Nascimento and DePrince (2016)), using time-independent orbitals, and their interpretation (Pedersen and Kvaal (2019); Pedersen et al. (2021)), including the very initial attempts (Schonhammer (1978); Hoodbhoy and Negele (1978, 1979)).

The TD-OCCDT scales as *O*(*N* ^8^) (*N*= the number of active orbitals), not ideally suited for applications to larger chemical systems. Therefore, we have developed a few lower cost methods in the TD-OCC framework (Pathak et al. (2020b,c,a, 2021)). We find triple excitations are necessary, including perfect optimization of the orbitals. Therefore, we are interested in developing affordable TD-OCC methods retaining a part of the triples. The most popular coupled-cluster method that treats the triple excitation amplitudes approximately is called CCSD(T) (Raghavachari et al. (1989); Watts et al. (1993)). Bozkaya *et al,* (Bozkaya and Schaefer (2012)) included various symmetric and asymmetric triple excitation corrections to their optimized double (OD) method.

In this communication, we report the formulation and implementation of the CCSD(T) method in the time-dependent optimized coupled-cluster framework, TD-OCCD(T). Following our previous works (Sato et al. (2018b); Pathak et al. (2020b,c, 2021)), we exclude single excitation amplitudes but optimize the orbitals according to time-dependent variational principle (TDVP). As the first application of this method, we study electron dynamics in Kr using intense near-infrared laser fields.

## 2 Methods

The second quantization representation of the Hamiltonian, including the laser field, is as follows,

$$\hat{H}=h_{\nu }^{\mu }\left(t\right){\hat{c}}_{\mu }^{†}{\hat{c}}_{\nu }+\frac{1}{2}u_{\nu \lambda }^{\mu \gamma }{\hat{c}}_{\mu }^{†}{\hat{c}}_{\gamma }^{†}{\hat{c}}_{\lambda }{\hat{c}}_{\nu }$$ (2)

where ${\hat{c}}_{\mu }^{†}$ $\left({\hat{c}}_{\mu }\right)$ represents a creation (annihilation) operator in a complete, orthonormal set of 2*n*_bas_ time-dependent spin-orbitals {*ψ*_*μ*_(*t*)}. *n*_bas_ is the number of basis functions used for expanding the spatial part of *ψ*_*μ*_, which, in the present real-space implementation, corresponds to the number of grid points, and

$$h_{\nu }^{\mu }\left(t\right)=\int dx_{1}\psi_{\mu }^{*}\left(x_{1}\right)\left[h_{0}+V_{\mathrm{ext}}\right]\psi_{\nu }\left(x_{1}\right),$$ (3)

$$u_{\nu \lambda }^{\mu \gamma }=\iint dx_{1}dx_{2}\frac{\psi_{\mu }^{*}\left(x_{1}\right)\psi_{\gamma }^{*}\left(x_{2}\right)\psi_{\nu }\left(x_{1}\right)\psi_{\lambda }\left(x_{2}\right)}{\left|r_{1}-r_{2}\right|},$$ (4)

where *x*_*i*_ = (***r***_*i*_, *σ*_*i*_) represents a composite spatial-spin coordinate. *h*_0_ is the field free one-electronic Hamiltonian and *V*_*ext*_ = *A*(*t*)*p*_*z*_ in the velocity gauge, *A*(*t*) = −*∫* ^*t*^ *E*(*t*′)*dt*′ is the vector potential, with *E*(*t*) being the laser electric field linearly polarized along the *z* axis.

The complete set of 2*n*_bas_ spin-orbitals (labeled with *μ*, *ν*, *γ*, *λ*) is divided into *n*_occ_ *occupied* (*o*, *p*, *q*, *r*, *s*) and 2*n*_bas_ − *n*_occ_ *virtual* spin-orbitals. The coupled-cluster (or CI) wavefunction is constructed only with occupied spin-orbitals, which are time-dependent in general, and virtual spin-orbitals form the orthogonal complement of the occupied spin-orbital space. The occupied spin-orbitals are classified into *n*_core_ *core* spin-orbitals, which are occupied in the reference Φ and kept uncorrelated, and *N* = *n*_occ_ − *n*_core_ *active* spin-orbitals (*t*, *u*, *v*, *w*) among which the active electrons are correlated. The active spin-orbitals are further split into those in the *hole* space (*i*, *j*, *k*, *l*) and the *particle* space (*a*, *b*, *c*, *d*), which are defined as those occupied and unoccupied, respectively, in the reference Φ. The core spin-orbitals can further be split into *frozen-core* space (*i*′′, *j*′′), fixed in time and the *dynamical-core* space (*i*′, *j*′), propagated in time (Sato and Ishikawa (2013)) (See. Figure 1 in Sato et al. (2018b) for a pictorial illustration).

**FIGURE 1:**
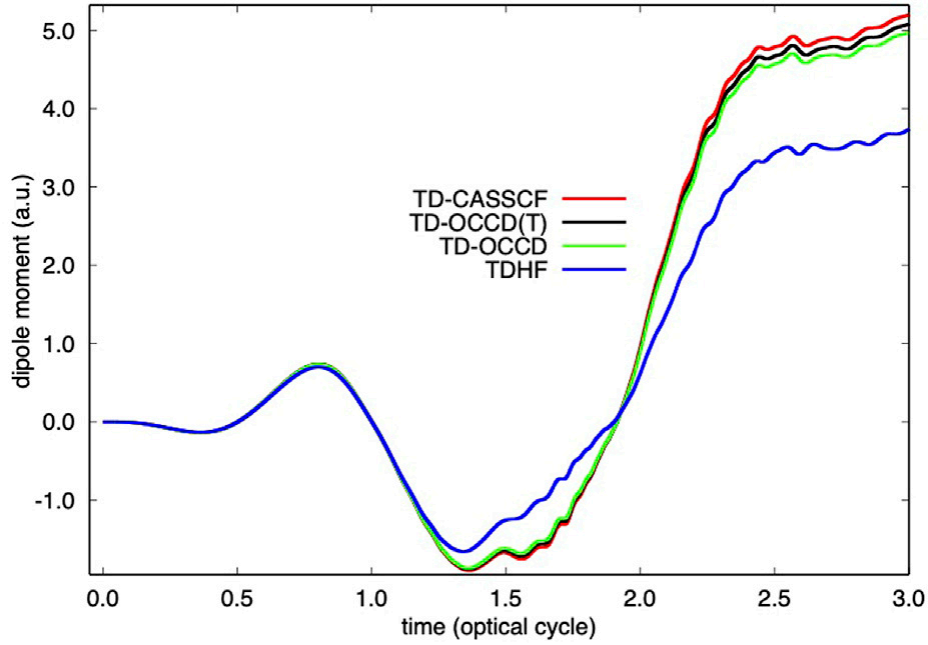
Time evolution of dipole moment of Kr irradiated by a laser pulse with a wavelength of 800 nm and a peak intensity of 2 × 10^14^ W/cm^2^ calculated with TDHF, TD-OCCD, TD-OCCD(T), and TD-CASSCF methods.

The real action formulation of the TDVP with orthonormal orbitals is our guiding principle, (Sato et al. (2018b))

$$S=Re\int_{t_{0}}^{t_{1}}Ldt=\frac{1}{2}\int_{t_{0}}^{t_{1}}\left(L+L^{*}\right)dt,$$ (5)

$$L=\left\langle \Phi |\left(1+\hat{\Lambda }\right)e^{-\hat{T}}\left(\hat{H}-i\frac{\partial }{\partial t}\right)e^{\hat{T}}|\Phi \right\rangle ,$$ (6)

$$\hat{T}={\hat{T}}_{2}+{\hat{T}}_{3}\cdots =\tau_{ij}^{ab}{\hat{E}}_{ij}^{ab}+\tau_{\mathrm{ijk}}^{\mathrm{abc}}{\hat{E}}_{\mathrm{ijk}}^{\mathrm{abc}}\cdots \;,$$ (7)

$$\hat{\Lambda }={\hat{\Lambda }}_{2}+{\hat{\Lambda }}_{3}\cdots =\lambda_{ab}^{ij}{\hat{E}}_{ab}^{ij}+\lambda_{\mathrm{abc}}^{\mathrm{ijk}}{\hat{E}}_{\mathrm{abc}}^{\mathrm{ijk}}\cdots \;,$$ (8)

where $\tau_{ij\cdots }^{ab\cdots }$ $\left(\lambda_{ab\cdots }^{ij\cdots }\right)$ are (de-)excitation amplitudes, and ${\hat{E}}_{ij\cdots }^{ab\cdots }={\hat{c}}_{a}^{†}{\hat{b}}_{b}^{†}\cdots {\hat{c}}_{j}{\hat{c}}_{i}$ . The stationary conditions, *δS* = 0, with respect to the variation of the parameters of the wavefunction ( $\delta \tau_{ij\cdots }^{ab\cdots }$ , $\delta \lambda_{ab\cdots }^{ij\cdots }$ , and *δψ*_*μ*_) gives us the corresponding equations of motions (EOMs), *δψ*_*μ*_ is orthonormality-conserving orbital variation.

For deriving the TD-OCCD(T) method, we first construct a fourth-order Lagrangian defined in Pathak et al. (2021). We make a further approximation to the Lagrangian and write separating it into two parts,

$$L_{\text{CCD}\left(T\right)}^{\left(4\right)}=L_{0}+\left\langle \Phi |\left(1+{\hat{\Lambda }}_{2}\right){\left[\left(\bar{f}+\hat{v}\right)e^{{\hat{T}}_{2}}\right]}_{c}|\Phi \right\rangle -i\lambda_{ab}^{ij}{\dot{\tau }}_{ij}^{ab}$$ (9a)

$$+\left\langle \Phi |{\hat{\Lambda }}_{2}{\left[\left(\bar{f}+\hat{v}\right){\hat{T}}_{3}\right]}_{c}|\Phi \right\rangle +\left\langle \Phi |{\hat{\Lambda }}_{3}{\left(\bar{f}{\hat{T}}_{3}\right)}_{c}|\Phi \right\rangle +\left\langle \Phi |{\hat{\Lambda }}_{3}{\left(\hat{v}{\hat{T}}_{2}\right)}_{c}|\Phi \right\rangle -i\lambda_{\mathrm{abc}}^{\mathrm{ijk}}{\dot{\tau }}_{\mathrm{ijk}}^{\mathrm{abc}},$$ (9b)

where $\bar{f}=\hat{f}-i\hat{X}$ , $\hat{f}=\left(h_{q}^{p}+v_{qj}^{pj}\right)\left\{{\hat{E}}_{q}^{p}\right\}$ , $\hat{v}=v_{qs}^{pr}\left\{{\hat{E}}_{qs}^{pr}\right\}/4$ , and $v_{qs}^{pr}=u_{qs}^{pr}-u_{sq}^{pr}$ , $\hat{X}=X_{\nu }^{\mu }{\hat{E}}_{\nu }^{\mu }$ , and $X_{\nu }^{\mu }=\left\langle \psi_{\mu }|{\dot{\psi }}_{\nu }\right\rangle$ is anti-Hermitian. The double amplitudes are obtained by making $L_{\text{CCD}\left(\text{T}\right)}^{\left(4\right)}$ of Eq. 9a stationary with respect to $\delta S/\delta \lambda_{ab}^{ij}\left(t\right)=0$ , $\delta S/\delta \tau_{ij}^{ab}\left(t\right)=0$ , the triples by making Eq. 9b stationary with respect to $\delta S/\delta \lambda_{\mathrm{abc}}^{\mathrm{ijk}}\left(t\right)=0$ , and $\delta S/\delta \tau_{\mathrm{ijk}}^{\mathrm{abc}}\left(t\right)=0$ ,

$$\begin{array}{ccc} i{\dot{\tau }}_{ij}^{ab} & = & v_{ij}^{ab}-p\left(ij\right){\bar{f}}_{j}^{k}\tau_{ik}^{ab}+p\left(ab\right){\bar{f}}_{c}^{a}\tau_{ij}^{cb}\\ & + & \frac{1}{2}v_{cd}^{ab}\tau_{ij}^{cd}+\frac{1}{2}v_{ij}^{kl}\tau_{kl}^{ab}+p\left(ij\right)p\left(ab\right)v_{ic}^{ak}\tau_{kj}^{cb}\\ & - & \frac{1}{2}p\left(ij\right)\tau_{ik}^{ab}\tau_{jl}^{cd}v_{cd}^{kl}+\frac{1}{2}p\left(ab\right)\tau_{ij}^{bc}\tau_{kl}^{ad}v_{cd}^{kl}\\ & + & \frac{1}{4}\tau_{kl}^{ab}\tau_{ij}^{cd}v_{cd}^{kl}+\frac{1}{2}p\left(ij\right)p\left(ab\right)\tau_{il}^{bc}\tau_{jk}^{ad}v_{cd}^{kl} \end{array}$$ (10)

$$\begin{array}{ccc} -i{\dot{\lambda }}_{ab}^{ij} & = & v_{ab}^{ij}-p\left(ij\right){\bar{f}}_{k}^{i}\lambda_{ab}^{kj}+p\left(ab\right){\bar{f}}_{a}^{c}\lambda_{cb}^{ij}\\ & + & \frac{1}{2}v_{ab}^{cd}\lambda_{cd}^{ij}+\frac{1}{2}v_{kl}^{ij}\lambda_{ab}^{kl}+p\left(ij\right)p\left(ab\right)v_{kb}^{cj}\lambda_{ac}^{ik}\\ & - & \frac{1}{2}p\left(ij\right)\lambda_{cd}^{ik}\tau_{kl}^{cd}v_{ab}^{jl}+\frac{1}{2}p\left(ab\right)\lambda_{bc}^{kl}\tau_{kl}^{cd}v_{ad}^{ij}\\ & + & \frac{1}{4}\lambda_{ab}^{kl}\tau_{kl}^{cd}v_{cd}^{ij}+\frac{1}{2}p\left(ij\right)p\left(ab\right)\lambda_{ac}^{jk}\tau_{kl}^{cd}v_{bd}^{il}\\ & - & \frac{1}{2}p\left(ij\right)\lambda_{ab}^{ik}\tau_{kl}^{cd}v_{cd}^{jl}\\ & + & \frac{1}{2}p\left(ab\right)\lambda_{bc}^{ij}\tau_{kl}^{cd}v_{ad}^{kl}+\frac{1}{4}\lambda_{cd}^{ij}\tau_{kl}^{cd}v_{ab}^{kl} \end{array}$$ (11)

$$\begin{array}{ccc} i{\dot{\tau }}_{\mathrm{ijk}}^{\mathrm{abc}} & = & p\left(k/ij\right)p\left(a/bc\right)v_{dk}^{bc}t_{ij}^{ad}-p\left(i/jk\right)p\left(c/ab\right)v_{jk}^{lc}t_{il}^{ab}\\ & - & p\left(k/ij\right){\bar{f}}_{k}^{l}\tau_{\mathrm{ijl}}^{\mathrm{abc}}+p\left(c/ab\right){\bar{f}}_{d}^{c}\tau_{\mathrm{ijk}}^{\mathrm{abd}}, \end{array}$$ (12)

$$\begin{array}{ccc} -i{\dot{\lambda }}_{\mathrm{abc}}^{\mathrm{ijk}} & = & p\left(k/ij\right)p\left(a/bc\right)v_{bc}^{dk}\lambda_{ad}^{ij}-p\left(c/ab\right)p\left(i/jk\right)v_{lc}^{jk}\lambda_{ab}^{ij}\\ & + & p\left(c/ab\right){\bar{f}}_{c}^{d}\lambda_{\mathrm{abd}}^{\mathrm{ijk}}-p\left(k/ij\right){\bar{f}}_{l}^{k}\lambda_{\mathrm{abc}}^{\mathrm{ijl}}\\ & + & p\left(i/jk\right)p\left(a/bc\right){\bar{f}}_{a}^{i}\lambda_{bc}^{jk}, \end{array}$$ (13)

where *p*(*μν*) and *p*(*μ*|*νγ*) are the permutation operators; *p*(*μν*)*A*_*μν*_ = *A*_*μν*_ − *A*_*νμ*_, and *p*(*μ*/*νγ*) = 1 − *p*(*μν*) − *p*(*μγ*).

The EOM for the orbitals can be written down in the following form Sato et al. (2016),

$$i|\dot{\psi_{p}}\rangle =\left(\hat{1}-\hat{P}\right)\hat{F}|\psi_{p}\rangle +i|\psi_{q}\rangle X_{p}^{q},$$ (14)

where $\hat{1}=\sum_{\mu }|\psi_{\mu }\rangle \langle \psi_{\mu }|$ is the identity operator within the space spanned by the given basis, $\hat{P}=\sum_{q}|\psi_{q}\rangle \langle \psi_{q}|$ is the projector onto the occupied spin-orbital space, and

$$\hat{F}|\psi_{p}\rangle =\hat{h}|\psi_{p}\rangle +{\hat{W}}_{s}^{r}|\psi_{q}\rangle P_{or}^{qs}{\left(D^{-1}\right)}_{p}^{o},$$ (15)

where *D* and *P* are Hermitialized one- (1RDM) and two- (2RDM) particle reduced density matrices defined in Sato et al. (2018b), and $W_{s}^{r}$ is the mean-field operator (Sato and Ishikawa (2013)). The matrix element $X_{p}^{q}$ includes orbital rotations among various subspaces. Non-redundant orbital rotations are determined by $i\left(\delta_{b}^{a}D_{i}^{j}-D_{b}^{a}\delta_{i}^{j}\right)X_{j}^{b}=F_{p}^{a}D_{i}^{p}-D_{p}^{a}F_{p}^{i*}-\frac{i}{8}{\dot{\tau }}_{\mathrm{ijk}}^{\mathrm{abc}}\lambda_{bc}^{jk}-\frac{i}{8}\tau_{\mathrm{ijk}}^{\mathrm{abc}}{\dot{\lambda }}_{bc}^{jk}$ . Redundant orbital rotations $\left\{X_{j^{\prime }}^{i^{\prime }}\right\}$ , $\left\{X_{j}^{i}\right\}$ , and $\left\{X_{b}^{a}\right\}$ can be arbitrary antiHermitian matrix elements. The general expressions for the RDMs are the same as in the TD-OCCDT(4) method (Pathak et al. (2021)).

## 3 Numerical results and discussion

Our numerical implementation has an interface with the Gaussian09 program (Frisch et al. (2009)) for checking ground state energy with the standard Gaussian basis results. We study BH molecule with double-*ζ* plus polarization (DZP). We have reported ground state energy computed by propagating in the imaginary time for OCCD and OCCD(T) methods in Table 1 and compared those with the optimized double and asymmetric triple excitation corrections for the orbital-optimized doubles method of Bozkaya *et al.*, Bozkaya and Schaefer (2012). We also compare our OCCD ground state energy result with Krylov *et al.*,Krylov et al. (1998) within the chosen active space of six electrons correlated among the six optimized active orbitals. We obtained a perfect agreement for all available values.

**TABLE 1:** Comparison of the ground state energy of BH (r_*e*_=2.4 bohr) molecule in DZP basis^a^.

Method	This work	References	
OCCD^b^	− 25.225 591 67	− 25.225 592	Bozkaya and Schaefer (2012)
OCCD(T)^b^	− 25.226 913 29	− 25.226 913	Bozkaya and Schaefer (2012)
OCCD^c^	− 25.178 285 70	− 25.178 286	Krylov et al. (1998)
OCCD(T)^c^	− 25.178 301 00		

a Gaussian09 program (Frisch et al. (2009)) is used to generate the required one-electron, two-electron, and overlap integrals, required for the imaginary time propagation of EOMs in the orthonormalized Gaussian basis. A convergence cut-off of 10^–15^ Hartree of energy difference is chosen in subsequent time steps.

b Six electrons correlated within the full basis set.

c Six electrons correlated within the six optimized active orbitals.

We have used a spherical-finite-element-discrete-variable representation (FEDVR) basis for representing orbital functions, Sato et al. (2016); Orimo et al. (2018) $\chi_{\mathrm{klm}}\left(r,\theta ,\psi \right)=\frac{1}{r}f_{k}\left(r\right)Y_{lm}\left(\theta ,\phi \right)$ where *Y*_*lm*_ and *f*_*k*_(*r*) are spherical harmonics and the normalized radial-FEDVR basis function, respectively. The expansion of the spherical harmonics continued up to the maximum angular momentum *L*_max_, and the radial FEDVR basis supports the range of radial coordinate 0 ≤ *r* ≤ *R*_max_, with cos^1/4^ mask function used as an absorbing boundary for avoiding unphysical reflection from the wall of the simulation box. We have used *l*_max_ = 72, and the FEDVR basis supporting the radial coordinate 0 < *r* < 300 using 78 finite elements each containing 25 DVR functions. The absorbing boundary is switched on at *r* = 180 in all our simulations. The Fourth-order exponential Runge-Kutta method (Hochbruck and Ostermann (2010)) is used to propagate the EOMs with 20000 time steps for each optical cycle. We run the simulations for a further 6,000 time steps after the end of the pulse. In all correlation calculations, eight electrons of 4*s*4*p* orbitals are considered as active and correlated among thirteen active orbitals. We report simulation results computed using a three-cycle laser pulse with a central wavelength of 800 nm having intensity 2 × 10^14^ W/cm^2^ and a period of *T* = 2*π*/*ω*_0_ ∼ 2.67 fs.

**FIGURE 2:**
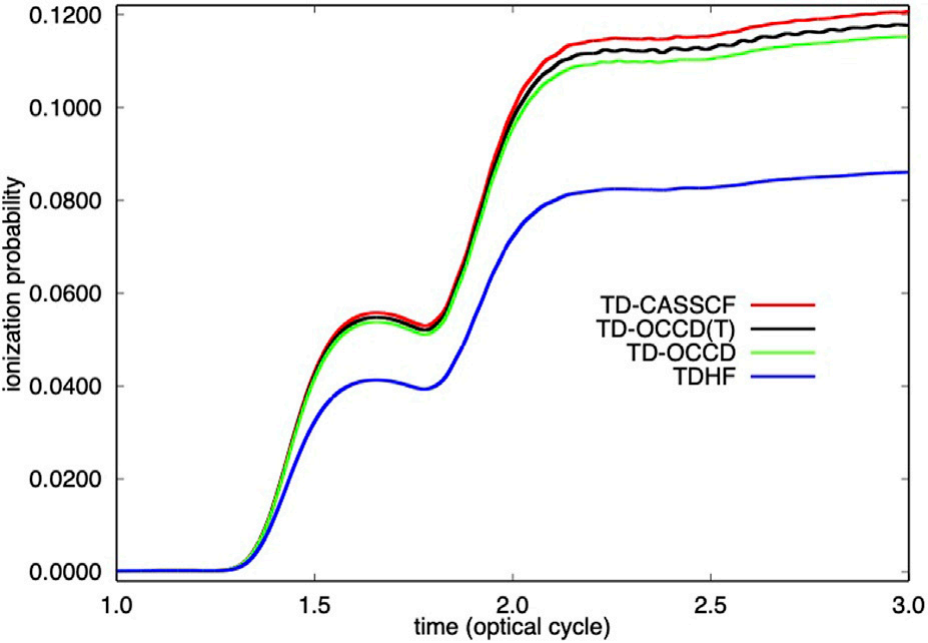
Time evolution of single ionization probability of Kr irradiated by a laser pulse with a wavelength of 800 nm and a peak intensity of 2 × 10^14^ W/cm^2^ calculated with TDHF, TD-OCCD, TD-OCCD(T), and TD-CASSCF methods.

We report the time evolution of dipole moment of Kr in Figure 1 and in Figure 2 single electron ionization probability. Time-dependent dipole moment is evaluated as a trace $\left\langle \psi_{p}|\hat{z}|\psi_{q}\right\rangle D_{p}^{q}$ using 1RDMs. For the single electron ionization probability, we computed the probability of finding an electron outside a sphere of a radius of 20 a.u. using RDMs defined in Refs. 19; 20; 37. We compare the results of TD-CASSCF, TD-OCCD(T), TD-OCCD, and TDHF methods.

We observe a substantial underestimation (both in Figure 1, and Figure 2) by the TDHF method due to the lack of correlation treatment. All correlation methods perform according to their ability to treat electron correlation. We also computed results using the TD-OCCDT method but not reported here since those results are not identifiable from the TD-CASSCF results within the graphical resolution.

**FIGURE 3:**
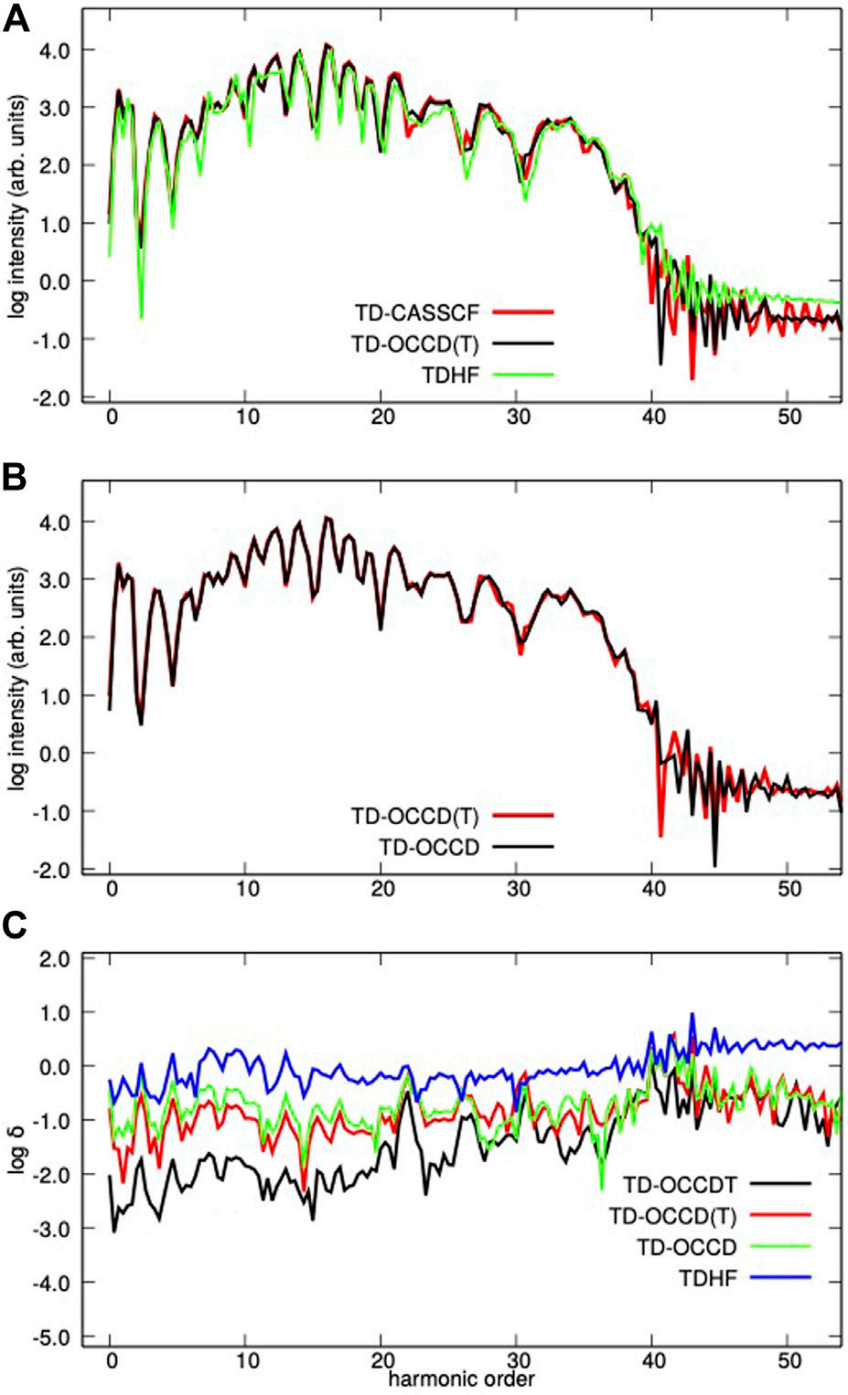
The HHG spectra **(A,B)** and the relative deviation **(C)** of the spectral amplitude from the TD-CASSCF spectrum from Kr irradiated by a laser pulse with a wavelength of 800 nm and a peak intensity of 2 × 10^14^ W/cm^2^ with various methods.

Next, we report high-harmonic generation in Figure 3. It is calculated by squaring the modulus *I*(*ω*) = |*a*(*ω*)|^2^ of the Fourier transform of the expectation value of the dipole acceleration with a modified Ehrenfest expression (Sato et al. (2016)). In panel (c) of Figure 3, we plot the absolute relative deviation (*δ*(*ω*), of the spectral amplitude *a*(*ω*) from the TD-CASSCF value for each method. All methods qualitatively predict similar HHG spectra with TDHF underestimates the spectral intensity. The relative deviation of results from TD-CASSCF ones follows the general trend TDHF > TD-OCCD > TD-OCCD(T) > TD-OCCDT, the same as what we observe for the time-dependent dipole moment and single ionization probability. We also simulated results with lower and higher intensity. However, the trend remains the same.

Finally, we make a tally of computational costs for all the methods considered in this article. All simulations performed using an Intel(R) Xeon(R) Gold 6,230 central processing unit (CPU) with 40 processors with a clock speed of 2.10 GHz, and report total simulations time in Table 2. Further, we report a reduction in the computational cost for various TD-OCC methods relative to the TD-CASSCF. We see a massive 63% cost reduction for the TD-OCCD(T) method, which is larger than for the TD-OCCDT method (58%), and a minimal increase from the TD-OCCD method.

**TABLE 2:** Comparison of the total simulation time^a^ (in min) spent for TD-CASSCF, TD-OCCDT, TDCCD(T), and TD-OCCD methods.

Method	Time (min)	Cost reduction (%)
TD-CASSCF	47303	…
TD-OCCDT	19697	58
TD-OCCD(T)	17504	63
TD-OCCD	17494	63

a Time spent for the simulation of Kr atom for 66000 time steps (0 ≤ *t* ≤ 3.3*T*) of a real-time simulation (*I*_0_ = 2 × 10^14^ W/cm^2^ and *λ* = 800 nm), using an Intel(R) Xeon(R) Gold 6230 CPU with 40 processors having a clock speed of 2.10 GHz.

## 4 Concluding remarks

We have reported the formulation and implementation of the TD-OCCD(T) method. As the first application, we employed this method to study laser-driven dynamics in Kr exposed to an intense near-infrared laser pulse. We observe a 63% cost reduction in comparison to the TD-CASSCF method without losing much accuracy. Therefore, we conclude that TD-OCCD(T) method will certainly be beneficial in exploring highly accurate *ab initio* simulations of electron dynamics in larger chemical systems.

## Data Availability

The raw data supporting the conclusion of this article will be made available by the authors, without undue reservation.
